# Impact of Immunosuppressive Therapy on the Performance of Latent Tuberculosis Screening Tests in Patients with Inflammatory Bowel Disease: A Systematic Review and Meta-Analysis

**DOI:** 10.3390/jpm12030507

**Published:** 2022-03-21

**Authors:** Chan Hyuk Park, Jung Ho Park, Yoon Suk Jung

**Affiliations:** 1 Department of Internal Medicine, Hanyang University Guri Hospital, Hanyang University College of Medicine, Guri 11923, Korea; yesable7@gmail.com; 2 Division of Gastroenterology, Department of Internal Medicine, Kangbuk Samsung Hospital, Sungkyunkwan University School of Medicine, Seoul 03181, Korea; jungho3.park@samsung.com

**Keywords:** interferon gamma release assay, tuberculin skin test, immunosuppressive therapy, inflammatory bowel disease

## Abstract

Screening for latent tuberculosis infection (LTBI) is mandatory before commencing tumor necrosis factor (TNF)-α inhibitor use. However, the impact of immunosuppressive therapy (IST), including corticosteroids and immunomodulators, on the performance of LTBI screening in patients with inflammatory bowel disease (IBD) has not been fully elucidated. We searched all relevant studies published before November 2021 that examined the performance of interferon γ release assays (IGRAs) and tuberculin skin tests (TSTs) in patients with IBD who received IST, using the Medline, EMBASE, and Cochrane Library databases. We performed meta-analyses of positive or indeterminate rates of IGRA or TST according to IST and calculated the concordance rates between IGRA and TST results. A total of 20 studies with 4045 patients were included in the meta-analysis. The IGRA-positive rate was lower in patients on IST than in those not on IST (odds ratio (OR) (95% confidence interval (CI)) = 0.55 (0.39–0.78)), whereas the IGRA-indeterminate rate was higher in patients on IST than in those not on IST (OR (95% CI) = 2.91 (1.36–6.24)). The TST-positive rate did not differ between the on-IST and not-on-IST groups (OR (95% CI) = 0.87 (0.51–1.50)). The concordance rate between IGRA and TST was 83.3% (95% CI, 78.5–88.1%). The IGRA-negative/TST-positive rate tended to be higher than that the IGRA-positive/TST-negative rate (9.5% vs. 5.8%, respectively), although the difference was not statistically significant. In conclusion, IGRA results were negatively affected by IST in patients with IBD, supporting requirements that IGRA should be performed before initiating IST. The use of both an IGRA and TST in patients with IBD on IST may improve the diagnosis rate of LTBI.

## 1. Introduction

Tuberculosis (TB) is an infectious disease that is one of the leading causes of death worldwide. The World Health Organization reported that in 2020, the incidence of TB reached 10 million people per year, and approximately 1.5 million people died from TB. It is estimated that one-third of the worldwide population is infected with *Mycobacterium tuberculosis* (MTB), of which 10% develop active tuberculosis (TB) infection and 90% remain a latent TB infection (LTBI), characterized by the presence of an immune response to MTB without symptoms or signs of TB disease [1]. However, LTBI can progress to active TB when there is an imbalance in host immune regulation caused by tumor necrosis factor (TNF)-α inhibitor therapy [1]. Because TNF-α plays an essential role in the host defense against MTB, its inhibition can increase the susceptibility to MTB and accelerate the reactivation of LTBI [2,3].

Although TNF-α inhibitors signal a new era in the treatment of inflammatory bowel disease (IBD), contributing greatly to the improvement in disease prognosis [4,5], the use of these agents is associated with a 2–8-fold increased risk of active TB [1]. This high risk of active TB in patients receiving TNF-α inhibitors is considered to be due to reactivation of LTBI rather than a new infection because most active TB cases develop within 3–4 months of TNF-α inhibitor initiation [6]. For these reasons, the guidelines strongly recommend screening for LTBI prior to initiation of TNF-α inhibitors [1].

Currently, there is no gold-standard test for diagnosing LTBI [1]. The traditional tuberculin skin test (TST) or the newer interferon γ release assay (IGRA) are used for LTBI diagnosis. TST results can be distorted by prior exposure to TB or Bacillus Calmette–Guérin (BCG) vaccination [7]. The IGRA is a blood test that measures interferon γ release by T cells after stimulation with antigens specific to MTB [8]. The commercially available IGRA tests include the QuantiFERON-TB Gold In-Tube test (QFT) that measures the amount of interferon γ in the supernatant of a cell suspension and the T-SPOT.TB test (T-SPOT), which measures the number of cells producing interferon γ [1]. Since the IGRA does not cross-react with the BCG vaccine, it is free from false-positive results in BCG-vaccinated individuals [8].

Although the IGRA has been reported to have a superior sensitivity and specificity for the diagnosis of LTBI than the TST [9,10], both the IGRA and TST may be negatively influenced by immunosuppressive therapy (IST) because IST can potentially inhibit T cells and impair the interferon γ response [11]. Indeed, several studies have reported lower IGRA-positive rates in IBD patients receiving IST, but there has been considerable variability between studies [12,13]. In 2012, a meta-analysis of nine studies (five abstracts and four published articles) was performed on the concordance of these tests and the impact on IST on their performance in patients with IBD [12]. More recently, a meta-analysis of 16 studies (5 abstracts, 1 letter, and 10 published articles) was conducted on the same topic [13]. However, the results of these meta-analyses were inconsistent and limited due to the small number of full-text studies included and the heterogeneity between studies. Moreover, the limited number of included studies prevented the evaluation of the effects of specific types of IST on IGRA and TST results. As several relevant studies have been published since then, it was necessary to update and complement the current evidence on this topic. In this study, we performed a systematic review and meta-analysis to assess the impact of IST on IGRA and TST results and the concordance of both tests in patients with IBD. In contrast to previous meta-analyses, our study considered the types of IST and the cutoff values for TSTs. The objective of our study is to help clinicians perform LTBI diagnostic tests more reliably in IBD patients.

## 2. Methods

### 2.1. Search Strategy

We searched for all relevant studies published between January 2000 and November 2021 that examined the performance of IGRA and TST results in IBD patients who received IST, using the Medline (available at https://pubmed.ncbi.nlm.nih.gov/; accessed date: 20 February 2022), EMBASE (available at https://ovidsp.ovid.com/; accessed date: 20 February 2022), and Cochrane Library (available at https://www.cochranelibrary.com/; accessed date: 20 February 2022) databases. The following search string was used: ((interferon gamma release assay) OR (interferon gamma release assay) OR (interferon gamma assay) OR (IGRA) OR (QuantiFERON) OR (QuantiFERON-TB) OR (QuantiFERONTB) OR (T-SPOT) OR (TSPOT-TB) OR (tuberculin skin test) OR (tuberculosis skin test) OR (TST) OR (PPD)) AND ((inflammatory bowel) OR (IBD) OR (Crohn) OR (Crohn’s) OR (ulcerative colitis)). Appendix A presents the detailed search strategies used for each database.

### 2.2. Inclusion/Exclusion Criteria

The inclusion criteria were as follows: (a) patients with IBD; (b) intervention—IST, including steroids, immunomodulators (e.g., azathioprine (AZA), 6-mercaptopurine (6-MP), and methotrexate (MTX)), and TNF-α inhibitors; (c) comparator—no IST; (d) outcome—IGRA- or TST-positive rates, IGRA-indeterminate rate, and concordance rate between IGRA and TST; and (e) study design—cohort or case–control studies. We excluded studies involving only pediatric patients with IBD to minimize heterogeneity of the meta-analysis results. Additionally, non-original studies, non-human studies, abstract-only publications, and non-English publications were excluded.

### 2.3. Study Selection

First, duplicates from multiple search engines were removed from the literature search results. We excluded irrelevant studies by reviewing the titles and abstracts according to the inclusion and exclusion criteria. Thereafter, we screened the full text of all remaining studies. Two investigators (C.H.P. and Y.S.J.) independently assessed the eligibility of individual studies. Any disagreements were resolved through discussion and consensus. If no agreement could be reached, a third investigator (J.H.P.) determined the final eligibility.

### 2.4. Quality Assessment

Two investigators (C.H.P. and Y.S.J.) independently assessed the quality of individual studies using the Newcastle–Ottawa Scale (NOS) [14]. They assigned scores to each study across three categories as follows: selection (4 points), comparability of the study groups (2 points), and ascertainment of exposure or outcome (3 points) [14]. Studies with a cumulative score of ≥7 points were considered high-quality studies.

### 2.5. Data Extraction

Data were extracted using a data extraction form (Excel spreadsheet) that had been developed in advance. Two investigators (C.H.P. and Y.S.J.) independently extracted the following information: first author, year of publication, study design, study period, country, publication language, study population, number of participants, demographics, definition of IST, type of IGRA method, cutoff value for the TST, IGRA- or TST-positive rate, IGRA-indeterminate rate, and concordance rate between the IGRA and TST.

### 2.6. Study Endpoints

The primary endpoint of this meta-analysis was the IGRA- or TST-positive rate according to the IST. The secondary endpoint was the IGRA-indeterminate rate according to the IST and the concordance rate between the IGRA and TST. For the primary or secondary endpoints related to the TST, only studies that used different cutoff values according to TB risk (e.g., 5 mm for patients on IST (or high-risk patients for TB) and 10 mm for patients who were not on IST (or low-risk patients for TB)) were considered because current CDC guidelines recommend different cutoff values based on certain risk factors for TB [15]. Studies that used a fixed cutoff value (5 mm) regardless of TB risk were considered in the sensitivity analysis.

### 2.7. Statistical Analysis

For the meta-analyses, pooled odds ratios (ORs) with 95% confidence intervals (CIs) were calculated. A random effects model was used. To identify the impact of the type of IST medication on the IGRA- or TST-positive rate, subgroup analyses were performed according to the IST medications (e.g., corticosteroids, immunomodulators, and TNF-α inhibitors). We assessed heterogeneity using two methods: Cochran’s *Q* test, wherein *p*-values < 0.1 were considered statistically significant for heterogeneity, and *I*^2^ statistics, wherein values of >50% were suggestive of significant heterogeneity [16]. We assessed publication bias qualitatively by inspecting funnel plots. Publication bias was assessed quantitatively using Egger’s test, wherein *p*-values < 0.1 were considered statistically significant [17]. Based on Cochrane recommendations, the funnel plot asymmetry test was not conducted when <10 studies were included [18]. Sensitivity analyses were performed according to the type of IGRA (QFT and T-SPOT), cutoff values for the TST, and BCG vaccination rate.

All *p*-values were two-tailed, and *p*-values < 0.05, except for heterogeneity tests and Egger’s test, were considered statistically significant. Analysis and reporting were performed in accordance with the Preferred Reporting Items for Systematic Reviews and Meta-Analyses guidelines [19]. All statistical analyses were conducted using Review Manager 5.3 (version 5.3.5; Cochrane Collaboration, Copenhagen, Denmark) and R (version 4.0.4; R Foundation for Statistical Computing, Vienna, Austria) with the metafor (version 2.4.0) (Wolfgang Viechtbauer, https://www.metafor-project.org/doku.php/updates#version_24-0_2020-03-19, accessed on 20 February 2022) [20] and meta (version 4.18.0) [21] packages.

## 3. Results

### 3.1. Study Selection and Characteristics

**Figure 1:**
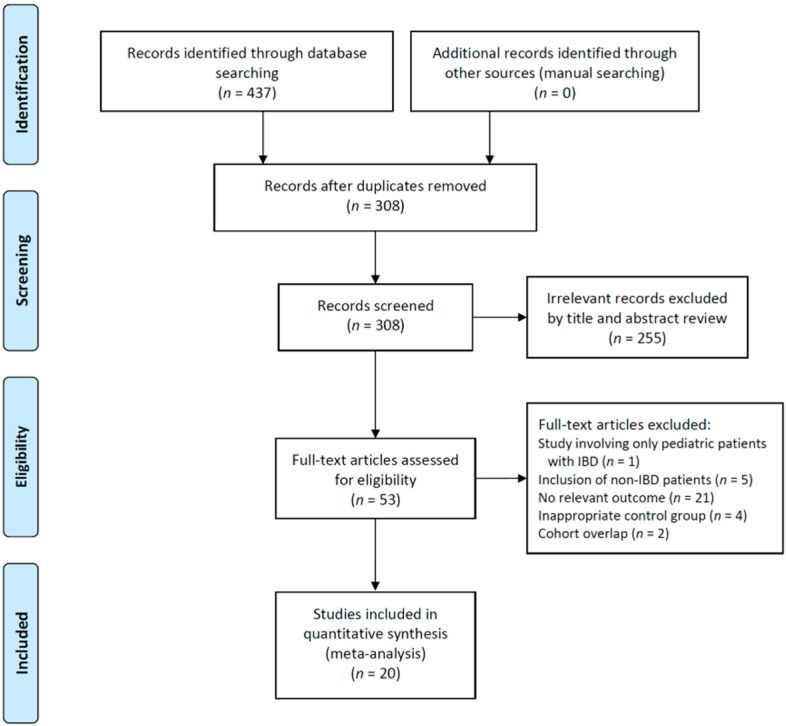
Study flow diagram. IBD—inflammatory bowel disease.

A total of 20 studies, including a total of 4045 patients, were included in the meta-analysis (Figure 1). The characteristics of the included studies are summarized in Table 1. The studies were published between 2008 and 2021 and included enrollment periods ranging from 2006 to 2017 [22,23,24,25,26,27,28,29,30,31,32,33,34,35,36,37,38,39,40,41]. Among the 20 included studies, 14 were prospective cohort studies [22,23,25,26,28,29,30,32,33,35,37,38,40,41], 5 were retrospective cohort studies [24,27,31,34,39], and the remaining 1 was a retrospective case–control study [36]. Fifteen studies reported IGRA-positive or IGRA-indeterminate rates according to IST [22,23,26,27,28,29,30,32,34,36,37,38,39,40,41]. Of these studies, 11 performed the IGRA using the QFT method [22,23,26,28,30,32,36,37,38,40,41] and 2 used the T-SPOT method [27,34], whereas 1 performed IGRA using both the QFT and T-SPOT [29]. The remaining study did not provide information about the type of IGRA method [39]. Additionally, 11 studies reported TST-positive rates according to the IST [22,23,26,28,29,32,35,37,38,40,41]. Among them, 7 used different cutoff values according to the risk of TB to determine a TST-positive result [22,23,26,32,37,38,41], whereas the other 4 used 5 mm as a cutoff value regardless of TB risk [28,29,35,40]. All the studies were considered to be of high quality by the NOS quality scores (Table 1).

### 3.2. IGRA-Positive or IGRA-Indeterminate Rates

**Figure 2:**
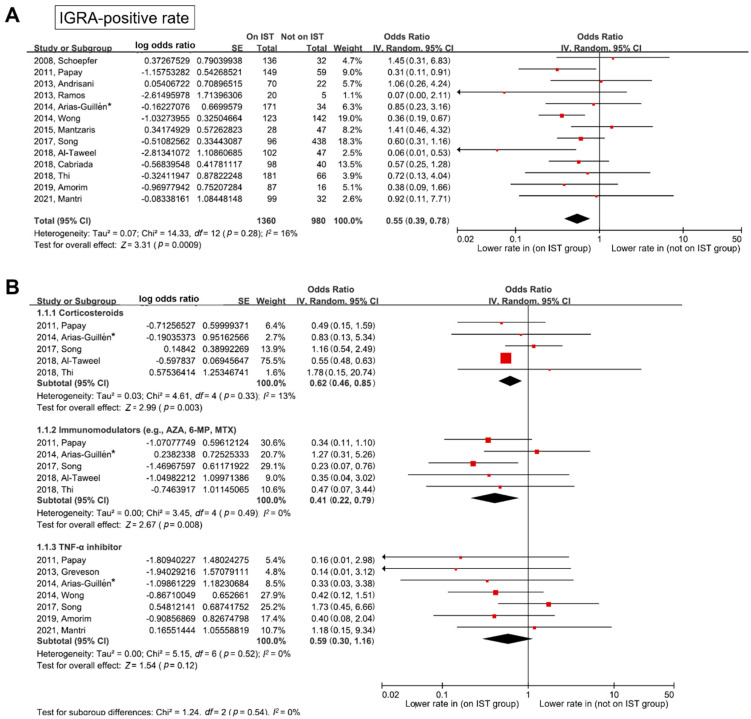
Forest plot for IGRA-positive rate between patients on IST and those not on IST. (**A**) Any IST, and (**B**) subgroup analysis according to IST. * In this study, both QFT and T-SPOT were performed. QFT data were included in the meta-analysis. IGRA—interferon γ release assay; IST—immunosuppressive treatment; QFT—QuantiFERON-TB Gold In-Tube test; T-SPOT—T-SPOT.TB test; AZA—azathioprine; 6-MP—6-mercaptopurine; MTX—methotrexate; TNF—tumor necrosis factor; SE—standard error; IV—inverse variance; CI—confidence interval; *df*—degrees of freedom.

The impact of IST on the of IGRA-positive rate is shown in Figure 2. The IGRA-positive rate was lower in patients on IST than in those not on IST (pooled OR (95% CI) = 0.55 (0.39–0.78)). Heterogeneity was not identified (degrees of freedom (*df*) = 12, *p* = 0.28, *I*^2^ = 16%). As shown in Figure 2B, in the subgroup analysis according to the type of IST medication, the IGRA-positive rate was lower in patients on IST with corticosteroids than in those not on IST (pooled OR (95% CI) = 0.62 (0.46–0.85)). In addition, the IGRA-positive rate was lower in patients on IST with immunomodulators than in those not on IST (pooled OR (95% CI) = 0.41 (0.22–0.79)). In the TNF-α inhibitor subgroup, the IGRA-positive rate tended to be lower in patients on IST than in those not on IST, although the difference was not statistically significant (pooled OR (95% CI) = 0.59 (0.30–1.16)). Heterogeneity was not identified in any of the above subgroups.

Figure S1 shows the impact of IST on the IGRA-indeterminate rate. The IGRA-indeterminate rate was higher in patients on IST than in those not on IST (pooled OR (95% CI) = 2.91 (1.36–6.24)). Heterogeneity was not identified (*df* = 5, *p* = 0.31, *I*^2^ = 16%). In the subgroup analyses, the use of corticosteroids was associated with a higher IGRA-indeterminate rate (pooled OR (95% CI) = 4.70 (1.86–11.83)). The IGRA-indeterminate rate tended to be higher in patients on IST with immunomodulators than in those not on IST, although the difference was not statistically significant (pooled OR (95% CI) = 3.61 (0.97–13.39)). However, the IGRA-indeterminate rate did not differ between patients on IST with TNF-α inhibitors and those not on IST (pooled OR (95% CI) = 1.28 (0.48–3.41)).

### 3.3. TST-Positive Rates

**Figure 3:**
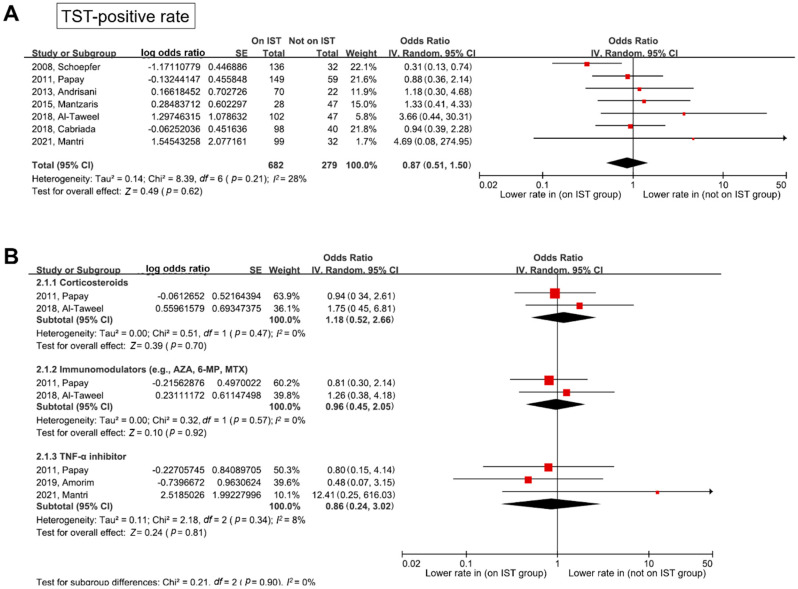
Forest plot for TST-positive rate between patients on IST and those not on IST. (**A**) Any IST, and (**B**) subgroup analysis according to IST. These analyses only included studies that used different cutoff values depending on TB risk according to the current guidelines (e.g., 5 mm for patients on IST (or high-risk patients for TB), and 10 mm for patients who were not on IST (or low-risk patients for TB)) [15]. TST—tuberculin skin test; IST—immunosuppressive treatment; TB—tuberculosis; AZA—azathioprine; 6-MP—6-mercaptopurine; MTX—methotrexate; TNF—tumor necrosis factor; SE—standard error; IV—inverse variance; CI—confidence interval; *df*—degrees of freedom.

Forest plots for the TST-positive rates in studies that used different cutoff values depending on TB risk according to the current guidelines [15], are shown in Figure 3. In contrast to the results of the IGRA-positive rate, the TST-positive rate was not associated with IST (pooled OR (95% CI) = 0.87 (0.51–1.50)) without significant heterogeneity (*df* = 6, *p* = 0.21, *I*^2^ = 28%). In the subgroup analyses according to the type of IST medications, the TST-positive rate did not differ between each IST medication group and the non-IST group (pooled OR (95% CI): corticosteroids, 1.18 (0.52–2.66); immunomodulators, 0.96 (0.45–2.05); and TNF-α inhibitors, 0.86 (0.24–3.02)). Heterogeneity was not identified in any of the subgroups.

### 3.4. Publication Bias

**Figure 4:**
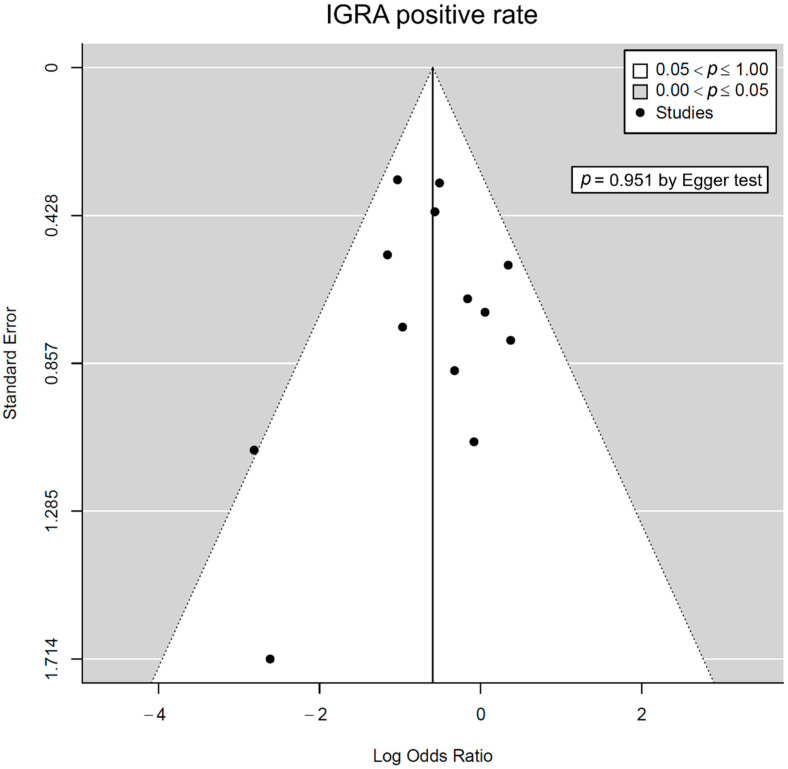
Funnel plots for IGRA-positive rate according to the use of IST. IGRA—interferon γ release assay; IST—immunosuppressive treatment.

Publication bias was assessed only in comparisons of the IGRA-positive rates because other comparisons included fewer than 10 individual studies. No significant asymmetry was observed in the funnel plots (Figure 4). Additionally, publication bias was not identified using Egger’s test (*p* = 0.951).

### 3.5. Sensitivity Analysis

Figure S2 shows the sensitivity analyses according to the type of IGRA method used (QFT and T-SPOT). As shown in Figure S2A, the IGRA-positive rate based on the QFT method was lower in patients on IST than in those not on IST (pooled OR (95% CI) = 0.54 (0.34–0.85)). The IGRA-positive rate based on the T-SPOT method tended to be lower in patients on IST than in those not on IST (pooled OR (95% CI) = 0.68 (0.39–1.20)), although the difference was not significant. The pooled OR of IST for the IGRA-positive rate did not significantly differ between the QFT and T-SPOT methods (*p* = 0.53).

Figure S3 shows the sensitivity analyses according to the inclusion of studies reporting TST positive rates based on a fixed cutoff value (5 mm) regardless of TB risk. When including both studies with different cutoff values according to the TB risk and those with a fixed cutoff value (5 mm), the pooled OR was 0.64 (95% CI, 0.40–1.04). Significant heterogeneity was identified in this analysis (*df* = 10, *p* = 0.02, *I*^2^ = 52%), which implies that the TST-positive rate depends on reliable cutoff values. In the meta-analysis of only studies with fixed cutoff values regardless of TB risk, the IGRA-positive rate was lower in patients on IST than in those not on IST (pooled OR (95% CI) = 0.33 (0.23–0.47)).

Figures S4 and S5 show the sensitivity analyses for IGRA-positive rate and TST-positive rate according to the BCG vaccination rates, respectively. As shown in Figure S4, the pooled OR for the IGRA-positive rate was not significantly different between studies with low BCG vaccination rates (<50%) and studies with high BCG vaccination rates (≥50%) (test for subgroup difference, *df* = 1, *p* = 0.53, *I*^2^ = 0%). Additionally, there was no significant difference in the pooled OR for the TST-positive rate between studies with low BCG vaccination rates and studies with high BCG vaccination rates (test for subgroup difference, *df* = 1, *p* = 0.17, *I*^2^ = 47%) (Figure S5).

### 3.6. Concordance Rates between IGRA and TST

**Figure 5:**
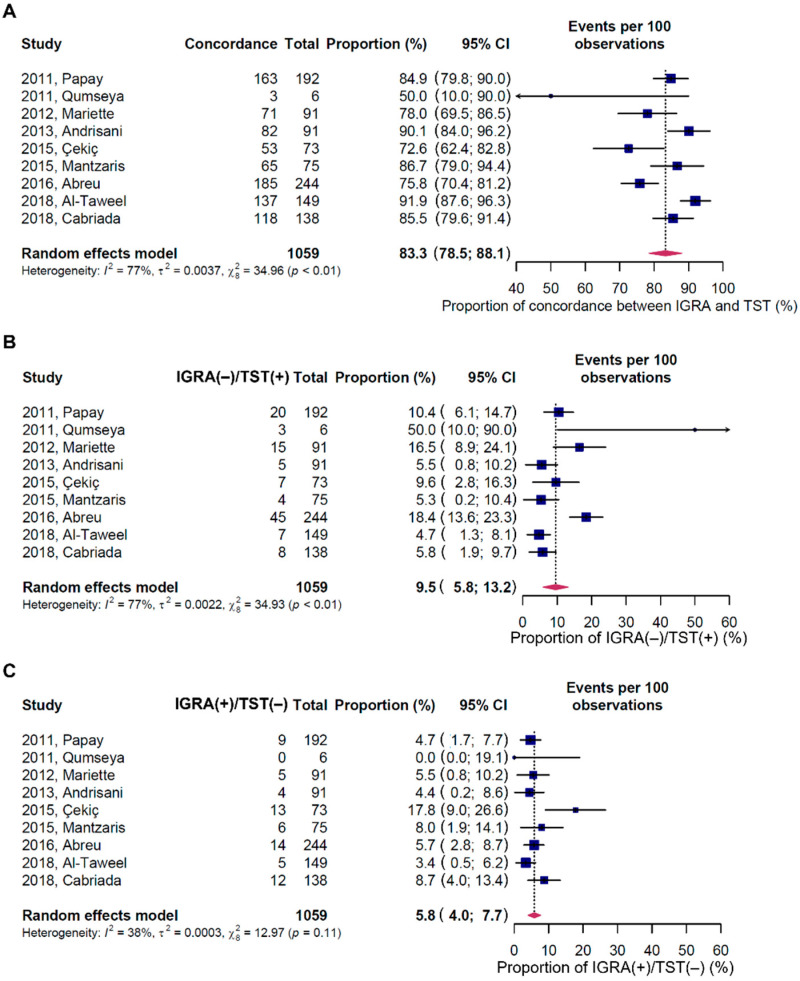
Concordance rates between IGRA and TST results. (**A**) Concordance, (**B**) IGRA-negative and TST-positive results, and (**C**) IGRA-positive and TST-negative results. The subscript number following χ^2^ indicates the degrees of freedom. IGRA—interferon γ release assay; TST—tuberculin skin test; CI—confidence interval.

The concordance rates between IGRA and TST results were analyzed in nine studies that provided both test results (Figure 5) [23,24,25,26,31,32,33,37,38]. In these analyses, only studies with different cutoff values of TST according to TB risk were considered.

The pooled concordance rate between the IGRA and TST was 83.3% (95% CI, 78.5–88.1%). Additionally, the pooled rate of IGRA-negative/TST-positive was 9.5% (95% CI, 5.8–13.2%), whereas that of IGRA-positive/TST-negative was 5.8% (95% CI, 4.0–7.7%). The rate of IGRA-negative/TST-positive tended to be higher than that of IGRA-positive/TST-negative, although the difference was not statistically significant.

## 4. Discussion

This is the first and largest meta-analysis to evaluate the performance of the IGRA and TST according to the type of IST in patients with IBD. Furthermore, this is the first meta-analysis to assess the impact of IST on indeterminate IGRA results and to consider the cutoff value for the TST. We identified several key findings: (a) IST (particularly corticosteroids and immunomodulators) decreases the IGRA-positive rate; (b) IST (particularly corticosteroids) increases the IGRA-indeterminate rate; and (c) TST results are not affected by IST if different cutoff values according to TB risk are used.

As mentioned in the introduction section, only two meta-analyses have evaluated the effect of IST on the IGRA and TST results in patients with IBD. An older meta-analysis, including studies up to June 2011, revealed that IGRA-positive and TST-positive rates were lowered by IST [12]. However, only four studies were available to calculate pooled estimates for assessing outcomes [12]. A more recent meta-analysis, including studies up to April 2018, did not find any significant effect of IST on both IGRA and TST results [13]. In this latter meta-analysis, the IGRA-positive rate tended to be lower in patients on IST, but the difference was not statistically significant (pooled OR (95% CI) = 0.57 (0.31–1.03)), and the TST-positive rate was not affected by IST (pooled OR (95% CI) = 1.14 (0.61–2.12)) [13]. However, both previous meta-analyses did not consider the specific type of IST and the cutoff value for the TST due to the limited number of studies included. Additionally, neither study performed a meta-analysis of IGRA-indeterminate results.

Another meta-analysis, including studies up to 2014, assessed the performance of the IGRA according to the type of IST in patients with autoimmune diseases [42]. This meta-analysis demonstrated that steroids, oral immunosuppressants, and TNF-α inhibitors were associated with a lower IGRA-positive rate [42]. However, given that this study did not focus on IBD and the IGRA results included 5 studies on IBD and 10 studies on rheumatologic diseases, it may be difficult to generalize the results of this meta-analysis to patients with IBD. This study also failed to conduct a subgroup analysis based on the type of IST in patients with IBD.

Summarizing previous meta-analyses, IST reduced the IGRA-positive rate, but the effect of IST on the TST was inconsistent. This may be because previous meta-analyses did not consider the TST cutoff value. We found that IST did not affect the TST-positive rate in the meta-analysis of studies with different cutoff values depending on TB risk according to the current guidelines (e.g., 5 mm for patients on IST and 10 mm for those not on IST) [15], whereas IST decreased the TST-positive rate in the meta-analysis of studies with fixed cutoff values (e.g., 5 mm for both patients on IST and not on IST). The reason for these results is that in studies that used fixed cutoff values, the TST-positive rate was higher in patients not on IST, and thus, the positive rate was relatively lower in those on IST. Based on the TST results, if the IGRA cutoff value was set differently depending on IST, that is, if the IGRA cutoff value was reduced for patients on IST, the diagnosis rate of LTBI in these patients may be increased. These efforts are needed to increase the detection rate of LTBI in patients with IBD receiving IST.

The significant negative impact of IST on the IGRA results suggests that IBD patients on IST are not completely free from the risk of TB reactivation, even if the IGRA result is negative. Cases that progressed to active TB despite a negative IGRA result have been occasionally reported. One review article reported that in the immunocompromised population, 13 of the 1999 (0.65%) QFT-negative patients and 26 of the 1273 (2.04%) T-SPOT-negative patients progressed to active TB [43]. Our meta-analysis results strongly support that an IGRA should be performed prior to initiation of corticosteroids and immunomodulators as well as prior to initiation of TNF-α inhibitors. Given that many IBD patients require corticosteroid treatment at the early stage of diagnosis and maintain remission with immunomodulators [44,45], it is ideal to perform an IGRA at the time of diagnosis of IBD. However, clinicians often encounter cases in which an IGRA is not performed at diagnosis but after IST has already started. In these unavoidable cases, it may be helpful to use an IGRA and TST together. The “either IGRA- or TST-positive” strategy may improve the sensitivity for detecting LTBI in IBD patients on IST. In fact, one study showed that employing this strategy before introducing TNF-α inhibitor therapy reduced the incidence of active TB by increasing the proportion of patients receiving LTBI treatment [46]. Several guidelines also recommend using both the IGRA and TST [1,47]. Since the IGRA is a convenient test that requires only a blood sample, does not cross-react with the BCG vaccine, and has been reported to have a higher sensitivity for the diagnosis of LTBI than TST [9,10], it may be desirable to perform an IGRA first, followed by a TST if the IGRA results are negative. Furthermore, our results in which the IGRA-negative/TST-positive rate was higher than the IGRA-positive/TST-negative rate (9.5% vs. 5.8%, respectively) support that an “IGRA followed by TST” strategy rather than a “TST followed by IGRA” strategy can further reduce the miss rate for LTBI detection. Additionally, an “IGRA followed by TST” strategy may be more appropriate in patients with a confirmed history of BCG vaccination or in countries with high BCG vaccination rates because TST results can be inaccurate for BCG-vaccinated patients.

Our meta-analysis provides more reliable evidence for the impact of IST on IGRA and TST results. Nonetheless, our study has some limitations. First, the definition of IST was slightly different between studies. To overcome this, we conducted subgroup analyses according to the types of IST, and no heterogeneity between studies was observed in both IGRA- and TST-positive rates. Second, BCG vaccination rates varied among studies, and this information was not available in six studies. Furthermore, the vaccination rates according to IST were unavailable. Accordingly, BCG vaccination could not be considered in the analysis of TST-positive rates. Third, TB prevalence/risk varies from country to country, but this was not taken into account in the analysis. Finally, in our meta-analysis, the positive rates, rather than the true positive or accuracy rates, were evaluated. The difference in IGRA- or TST-positive rates according to IST may be biased if the prevalence of LTBI differed between patients on IST and those who were not on IST. However, this is an unavoidable limitation because there is no gold-standard test for the diagnosis of LTBI [1]. Considering that significant heterogeneity was not identified across the included studies, potential bias caused by different levels of prevalence of LTBI between patients on IST and those who were not on IST is likely to be minimal.

In conclusion, this meta-analysis demonstrated a marked negative impact of corticosteroids and immunomodulators on the IGRA results in patients with IBD, suggesting that an IGRA may be unreliable for the diagnosis of LTBI in patients receiving these therapies. Our results strongly support that an IGRA should be performed prior to the initiation of IST. The optimal timing of this IGRA is at the time of diagnosis. However, if screening tests for LTBI are required in a situation where IST has already started, the use of both the IGRA and TST (“either test positive” strategy) may improve the detection of LTBI.

## Figures and Tables

**Table 1:** Baseline characteristics of included studies.

Publication Year, First Author [Reference Number]	Study Design	Study Period	Country	Study Population	Number of Participants	Age, Year	Male, %	BCG Rate, %	Definition of IST	Medication for IST (%) ^a^	IGRA Method	TST Cutoff, mm	Newcastle–Ottawa Scale (Selection/Comparability/Outcome)
2008, Schoepfer [22]	Prospective cohort	2006–2007	Switzerland	CD: 114 UC: 44 Indeterminate colitis:10	168	Mean 41	49.4	70.2	Prednisone ≥15 mg/day (or equivalent dose of corticosteroid) for ≥1 month, AZA ≥ 2 mg/kg, 6-MP ≥ 1 mg/kg, MTX ≥ 15 mg/week, or TNF-α inhibitor	AZA (39.9) 6-MP (11.9) MTX (16.7) Steroids (25.0) IFX (14.9)	QFT	5 (contact with TB, changes on chest X-ray, organ transplant, immunosuppressed state), 10 (high risk for TB), 15 (low risk for TB)	4/1/3
2011, Papay [23]	Prospective cohort	2006–2009	Austria	CD: 152 UC: 56	208	Mean 36.6	48.6	100.0	Steroids at any dose ≥2 weeks, thiopurines or MTX ≥ 3 months, or TNF-α inhibitor within the last 12 weeks	Thiopurines or MTX (47.1) Steroids (33.7) TNF-α inhibitor (8.7) Combination of different ISTs (16.8)	QFT	5 (on IST), 10 (not on IST)	4/1/3
2011, Qumseya [24]	Retrospective cohort	N/A	USA	CD: 296 UC: 44	340	Mean 41	45.6	N/A	Steroids, AZA, 6-MP, thioguanine, MTX, or TNF-α inhibitor	MTX (12.1) AZA (17.6) 6-MP (8.5) Thioguanine (0.3) Mycophenolate mofetil (1.2) Lenalidomid (0.3) TNF-α inhibitor (58.2)	QFT	5 (on IST), 10 (not on IST)	4/1/3
2012, Mariette [25]	Prospective cohort	N/A	France	CD: 91	91	Median 36	47.2	65.9	Corticosteroids or immunosuppressants	Corticosteroids (33.0) Other immuno-suppressants (44.0) Combination (61.5)	QFT or T-SPOT	5	4/1/3
2013, Andrisani [26]	Prospective cohort	2008–2010	Italy	CD: 60 UC: 32	92	Mean 39.6	50.0	1.1	Prednisone ≥ 20 mg/day (or equivalent dose of corticosteroid) for ≥2 weeks, thiopurines (2–2.5 mg/kg/day) or MTX (10–15 mg/week) ≥3 months, or TNF-α inhibitor within the last 12 weeks	AZA (41.3) MTX (2.2) Steroids (32.6)	QFT	5 (on IST), 10 (not on IST)	4/1/3
2013, Greveson [27]	Retrospective cohort	2008–2010	United Kingdom	CD: 102 UC: 16 Indeterminate colitis: 7	125	Range 27–45	51.2	87.2	Steroids > 20 mg/day, AZA, 6-MP, MTX, or TNF-α inhibitor	Corticosteroid (17.6) Thiopurines (52.8) TNF-α inhibitor (28.0)	T-SPOT	Not applicable	4/1/3
2013, Ramos [28]	Prospective cohort	2009–2011	Spain	IBD: 25	25	Median 30	60.0	8.0	Corticosteroids ≥ 5 mg/day for >4 weeks), cyclosporine ≥ 2.5 mg/kg/day, AZA ≥ 1 mg/kg/day, leflunomide 20 mg/day, or MTX ≥ 7.5 mg/week	Corticosteroid (20.0) AZA (76.0)	QFT	5	4/1/3
2014, Arias-Guillén [29]	Prospective cohort	2009–2011	Spain	CD: 157 UC: 42 Indeterminate colitis: 6	205	Mean 44	50.2	89.8	Corticosteroids within the last 2 weeks, immunomodulators (AZA, 6-MP, or MTX) within the last 3 months, or TNF-α inhibitor within the last 12 weeks	Corticosteroids (13.2) Immunomodulators (31.2) TNF-α inhibitor (15.6) Combination of different ISTs (23.4)	QFT and T-SPOT	5	4/1/3
2014, Wong [30]	Prospective cohort	N/A	China	CD: 128 UC: 136 Indeterminate colitis: 4	268	Mean 43	59.7	73.1	Prednisone 15 mg/day (or equivalent dose of corticosteroid) for ≥1 month, AZA, 6-MP, MTX, or TNF-α inhibitor	Corticosteroid, AZA, 6-MP, or MTX (46.4) TNF-α inhibitor (7.5)	QFT	5	4/1/3
2015, Çekiç [31]	Retrospective cohort	2007–2014	Turkey	CD: 51 UC: 25	76	Mean 42	69.7	N/A	Prednisolone 20 mg/day (or equivalent dose of corticosteroid) for ≥2 weeks, AZA for ≥3 months, or TNF-α inhibitor	Corticosteroid (18.4) AZA (53.9) TNF-α inhibitor (100.0)	QFT	5	4/1/3
2015, Mantzaris [32]	Prospective cohort	2008–2010	Greece	CD: 53 UC: 22	75	Median 37	56.0	63.4	Thiopurines	Thiopurines 37.3	QFT	5 (on IST), 10 (not on IST)	4/1/3
2016, Abreu [33]	Prospective cohort	2012–2015	Portugal	CD: 203 UC: 47	250	Median 36	43.6	100.0	Steroids ≥ 2 weeks, thiopurines ≥ 2 months, MTX ≥ 2 months, cyclosporine ≥ 2 months, or TNF-α inhibitor	Steroids (10.0) AZA (30.0) Steroids + AZA (18.0) MTX (0.4) Steroids + AZA + MTX or cyclosporine (0.8) TNF-α inhibitor (8.0) TNF-α inhibitor + other ISTs (17.6)	QFT	5	4/1/3
2017, Song [34]	Retrospective cohort	2011–2016	China	CD: 187 UC: 300 Indeterminate colitis: 47	534	Mean 39.6	63.3	N/A	Prednisone ≥ 20 mg/day (or equivalent dose of corticosteroid) for ≥2 weeks within the last 3 months, any dose of AZA, MTX, thalidomide, or cyclosporine within the last 3 months, or TNF-α inhibitor within the last 12 weeks	N/A	T-SPOT	Not applicable	4/2/3
2017, Taxonera	Prospective cohort	N/A	Spain	CD: 349 UC: 223	580	Mean 42.6	55.3	22.4	Steroid at any dose for ≥2 weeks, AZA for ≥2 months, 6-MP for ≥2 months, MTX for ≥2 months, or TNF-α inhibitor	Steroids (6.5) AZA, 6-MP, MTX (15.4) TNF-α inhibitor (10.1) Combination of different ISTs (37.5)	Not applicable	5	4/1/3
2017, Vajravelu	Retrospective case–control	2009–2014	USA	CD: 163 UC: 68 Indeterminate colitis: 9	240	<45 years, 59.2% 45–65 years, 31.3% >65 years, 9.6%	46.3	N/A	Corticosteroids, immunomodulators (AZA, 6-MP, or MTX), or TNF-α inhibitor	Corticosteroids (46.3) Immunomodulators (21.3) TNF-α inhibitor (17.5)	QFT	Not applicable	3/2/3
2018, Al-Taweel	Prospective cohort	2010–2014	Canada	CD: 127 UC: 21 Indeterminate colitis: 1	149	Mean 38.1	51.7	17.5	Corticosteroids ≥ 15 mg for >4 weeks, thiopurines, or MTX	Corticosteroids (43.6) Thiopurines (30.9) Methotrexate (6.7)	QFT	5 (low risk for TB), 10 (high risk for TB)	4/2/3
2018, Cabriada	Prospective cohort	N/A	Spain	CD: 112 UC: 26	138	Median 39.5	55.8	N/A	Prednisone ≥ 15 mg/day (or equivalent dose of corticosteroids) for ≥1 month, immunomodulators, including AZA, 6-MP, and MTX, for ≥3 months, or TNF-α inhibitor ≥ 3 months	Corticosteroids (23.2) Immunomodulators (29.7) TNF-α inhibitor (0.7) Combination of different ISTs (17.4)	QFT	5 (on IST), 10 (not on IST)	4/2/3
2018, Thi	Retrospective cohort	2007–2015	United Kingdom	CD: 173 UC: 74	247	Median 34	53.8	N/A	Corticosteroids, thiopurines, MTX, tacrolimus, or mycophenolate	Corticosteroids (7.7) Thiopurines (49.0) MTX (3.6) Tacrolimus or mycophenolate (2.8) Combination of different ISTs (10.1)	N/A	Not applicable	4/1/3
2019, Amorim	Prospective cohort	2015–2017	Brazil	CD: 83 UC: 20	103	Mean 37.6	43.7	98.1	Prednisone ≥ 20 mg/day for ≥2 weeks, immunomodulators (AZA, 6-MP, or MTX) within the last 3 months, or TNF-α inhibitor within the last 3 months	Corticosteroids (4.9) Immunomodulators (16.5) TNF-α inhibitor (10.7) Combination of different ISTs (52.4)	QFT	5	4/1/3
2021, Mantri	Prospective cohort	N/A	India	CD: 121 UC: 10	131	Mean 36.0	55.0	22.9	Steroids for ≥2 weeks, thiopurines for ≥2 months, MTX for ≥2 months, cyclosporine for ≥2 months, or TNF-α inhibitor	Steroids (69.5) AZA (50.4) TNF-α inhibitor (34.4)	QFT	5 (on IST), 10 (not on IST)	4/2/3

^a^ Percentage is calculated based on the number of all participants in each study. IST—immunosuppressive treatment; BCG—Bacillus Calmette–Guérin; IGRA—interferon γ release assay; TST—tuberculin skin test; IBD—inflammatory bowel disease; CD—Crohn’s disease; UC—ulcerative colitis; AZA—azathioprine; 6-MP—6-mercaptopurine; MTX—methotrexate; TNF—tumor necrosis factor; IFX—infliximab; QFT—QuantiFERON-TB Gold In-Tube test; T-SPOT—T-SPOT.TB test; TB—tuberculosis; LTBI—latent tuberculosis infection; N/A—not available.

## Data Availability

All relevant data are included in the study and Supplementary Information.

## Supplementary Materials

The following are available online at https://www.mdpi.com/article/10.3390/jpm12030507/s1, Figure S1: Forest plot for IGRA-indeterminate rates between patients on IST and those not on IST, Figure S2: Sensitivity analysis for IGRA-positive rates according to type of IGRA test, Figure S3: Sensitivity analysis for TST-positive rate, Figure S4: Sensitivity analysis for IGRA-positive rates according to BCG vaccination rate (<50% vs. ≥50%), Figure S5: Sensitivity analysis for TST-positive rate according to BCG vaccination rate (<50% vs. ≥50%).

## Appendix A. Detailed Search Strategy

### MEDLINE (Search Interface: PubMed)

((interferon gamma release assay[tw]) OR (interferon gamma release assay[tw]) OR (interferon gamma assay[tw]) OR IGRA[tw] OR QuantiFERON[tw] OR QuantiFERON-TB[tw] OR QuantiFERONTB[tw] OR T-SPOT[tw] OR TSPOT-TB[tw] OR (T SPOT TB[tw]) OR (tuberculin skin test[tw]) OR (tuberculosis skin test[tw]) OR TST[tw] OR PPD[tw]) AND ((inflammatory bowel[tw]) OR IBD[tw] OR Crohn[tw] OR Crohn’s[tw] OR (ulcerative colitis[tw])) AND (“2000/01/01”[Date-Publication]: “3000”[Date-Publication]).

EMBASE (Search interface: Ovid)

1: (interferon gamma release assay or interferon gamma release assay or interferon gamma assay or IGRA or QuantiFERON or QuantiFERON-TB or QuantiFERONTB or T-SPOT or TSPOT-TB or tuberculin skin test or tuberculosis skin test or TST or PPD) and (inflammatory bowel or IBD or Crohn or Crohn’s or ulcerative colitis).ab,ti.
2: Limit 1 to (english language and embase and yr = “2000–Current”).

Cochrane library

#1: ‘interferon gamma release assay’ or ‘interferon-gamma release assay’ or ‘interferon gamma assay’ or IGRA or QuantiFERON or QuantiFERON-TB or QuantiFERONTB or T-SPOT or TSPOT-TB or ‘tuberculin skin test’ or ‘tuberculosis skin test’ or TST or PPD.
#2: ‘inflammatory bowel’ or IBD or Crohn or Crohn’s or ‘ulcerative colitis’.
#3: #1 and #2 (with Publication Year from 2000 to 2021, in Trials).
