# Supplementary material for: Impact of Immunosuppressive Therapy on the Performance of Latent Tuberculosis Screening Tests in Patients with Inflammatory Bowel Disease: A Systematic Review and Meta-Analysis
Source: J Pers Med. 2022 Mar 21;12(3):507. doi: 10.3390/jpm12030507 (PMC8953543; doi:10.3390/jpm12030507)
Supplement: Supplementary file 1 [file jpm-12-00507-s001.zip › jpm-1626999-supplementary.pdf]

Supplementary figures

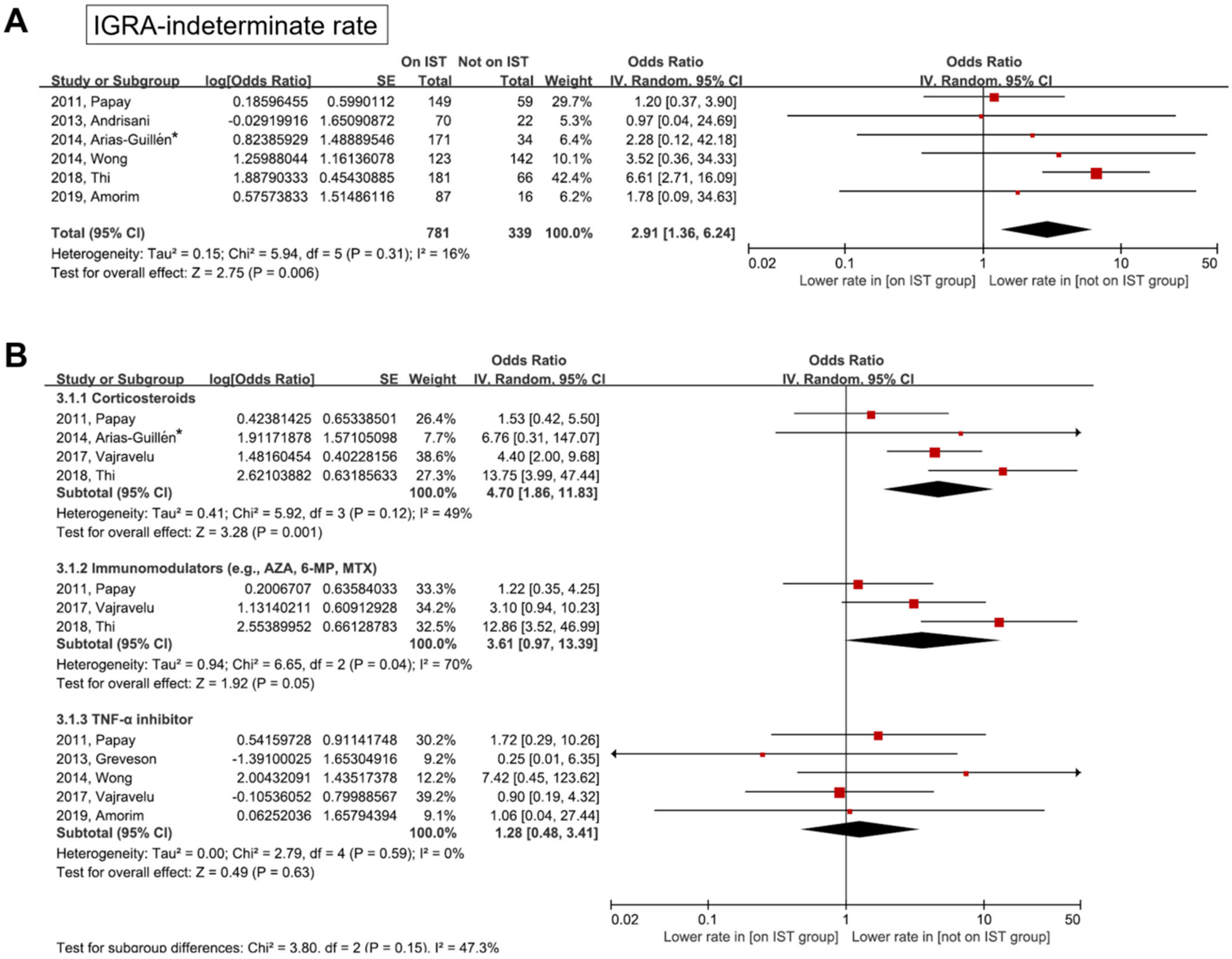

Figure S1. Forest plot for IGRA-indeterminate rates between patients on IST and those not on IST. (A) Any IST, and (B) Subgroup analysis according to IST.

*In this study, both QFT and T-SPOT were performed. QFT data were included in the meta-analysis.

IGRA, interferon γ release assay; IST, immunosuppressive treatment; QFT, QuantiFERON-TB Gold In-Tube test; T-SPOT, T-SPOT.TB test; AZA, azathioprine; 6-MP, 6-mercaptopurine; MTX, methotrexate; TNF, tumor necrosis factor; SE, standard error; IV, inverse variance; CI, confidence interval; df, degrees of freedom.

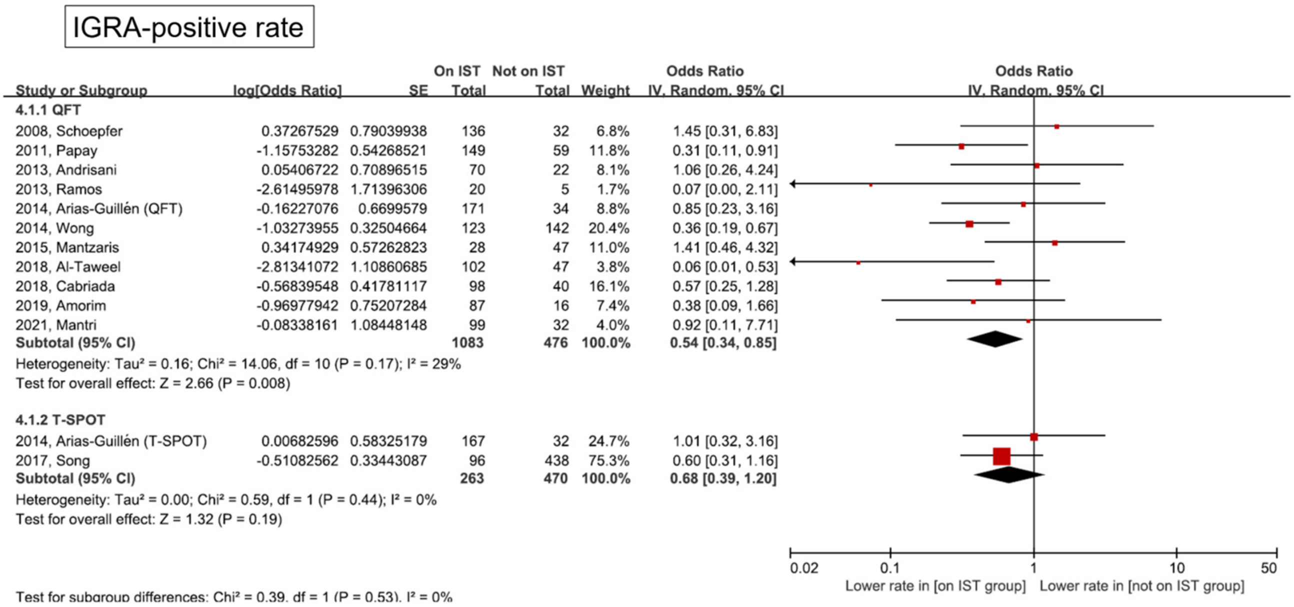

Figure S2. Sensitivity analysis for IGRA-positive rates according to type of IGRA test. (A) QFT, and (B) T-SPOT

IGRA, interferon γ release assay; IST, immunosuppressive treatment; QFT, QuantiFERON-TB Gold In-Tube test; T-SPOT, T-SPOT.TB test; SE, standard error; IV, inverse variance; CI, confidence interval; df, degrees of freedom.

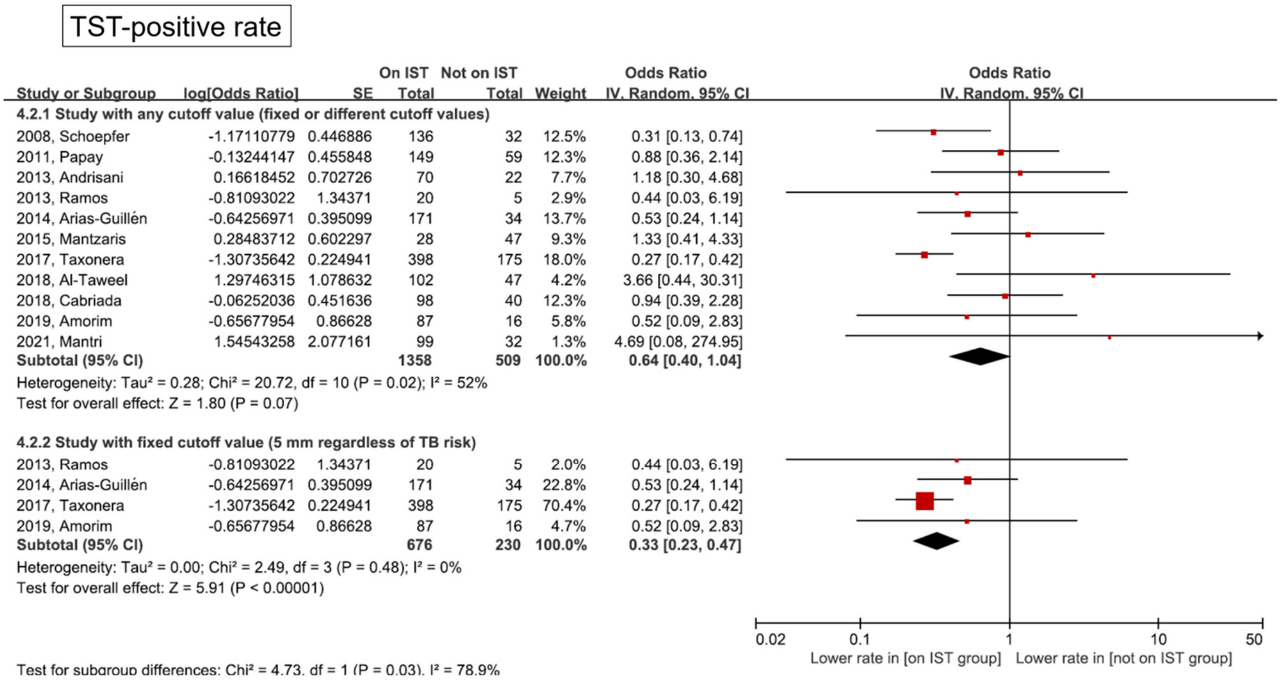
TST-positive rate

Study or Subgroup	log[Odds Ratio]	SE	On IST Total	Not on IST Total	Weight	Odds Ratio IV, Random, 95% CI
**4.2.1 Study with any cutoff value (fixed or different cutoff values)**						
2008, Schoepfer	-1.17110779	0.446886	136	32	12.5%	0.31 [0.13, 0.74]
2011, Papay	-0.13244147	0.455848	149	59	12.3%	0.88 [0.36, 2.14]
2013, Andrisani	0.16618452	0.702726	70	22	7.7%	1.18 [0.30, 4.68]
2013, Ramos	-0.81093022	1.34371	20	5	2.9%	0.44 [0.03, 6.19]
2014, Arias-Guillén	-0.64256971	0.395099	171	34	13.7%	0.53 [0.24, 1.14]
2015, Mantzaris	0.28483712	0.602297	28	47	9.3%	1.33 [0.41, 4.33]
2017, Taxonera	-1.30735642	0.224941	398	175	18.0%	0.27 [0.17, 0.42]
2018, Al-Taweel	1.29746315	1.078632	102	47	4.2%	3.66 [0.44, 30.31]
2018, Cabriada	-0.06252036	0.451636	98	40	12.3%	0.94 [0.39, 2.28]
2019, Amorim	-0.65677954	0.86628	87	16	5.8%	0.52 [0.09, 2.83]
2021, Mantri	1.54543258	2.077161	99	32	1.3%	4.69 [0.08, 274.95]
**Subtotal (95% CI)**			**1358**	**509**	**100.0%**	**0.64 [0.40, 1.04]**
Heterogeneity: Tau² = 0.28; Chi² = 20.72, df = 10 (P = 0.02); I² = 52%						
Test for overall effect: Z = 1.80 (P = 0.07)						
**4.2.2 Study with fixed cutoff value (5 mm regardless of TB risk)**						
2013, Ramos	-0.81093022	1.34371	20	5	2.0%	0.44 [0.03, 6.19]
2014, Arias-Guillén	-0.64256971	0.395099	171	34	22.8%	0.53 [0.24, 1.14]
2017, Taxonera	-1.30735642	0.224941	398	175	70.4%	0.27 [0.17, 0.42]
2019, Amorim	-0.65677954	0.86628	87	16	4.7%	0.52 [0.09, 2.83]
**Subtotal (95% CI)**			**676**	**230**	**100.0%**	**0.33 [0.23, 0.47]**
Heterogeneity: Tau² = 0.00; Chi² = 2.49, df = 3 (P = 0.48); I² = 0%						
Test for overall effect: Z = 5.91 (P < 0.00001)						

Test for subgroup differences: Chi² = 4.73, df = 1 (P = 0.03), I² = 78.9%

Figure S3. Sensitivity analysis for TST-positive rate. (A) All studies with any cutoff values of TST (both fixed cutoff value and different cutoff value according to TB risk), and (B) Studies with fixed cutoff value (5 mm) regardless of TB risk
TST, tuberculin skin test; IST, immunosuppressive treatment; TB, tuberculosis; SE, standard error; IV, inverse variance; CI, confidence interval; df, degrees of freedom.

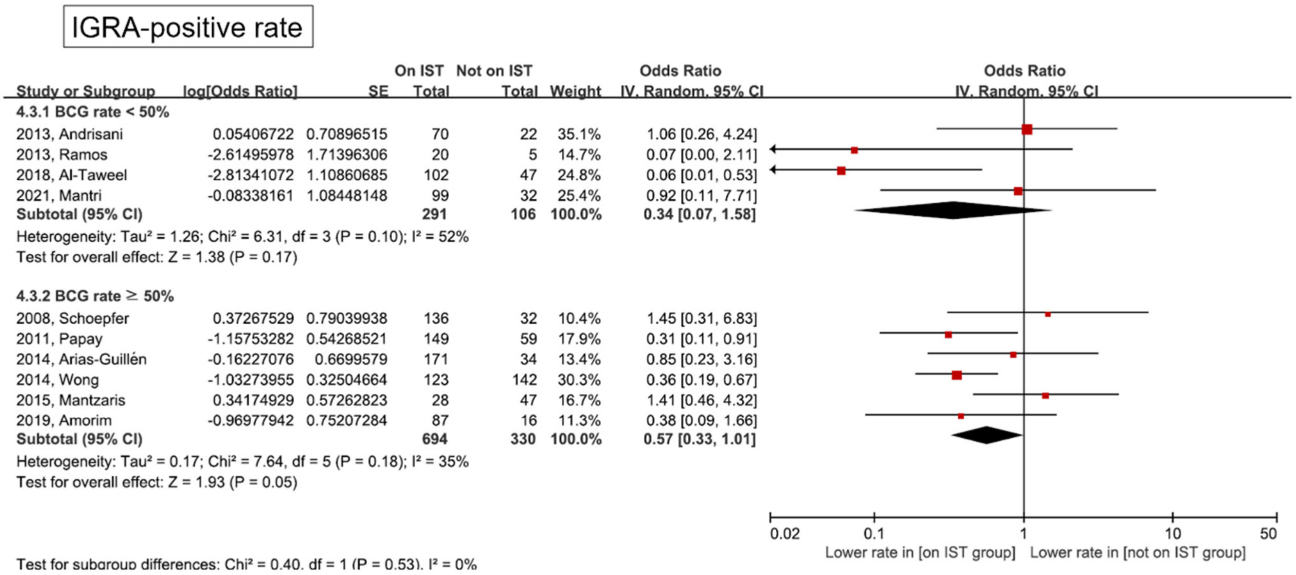

Figure S4. Sensitivity analysis for IGRA-positive rates according to BCG vaccination rate (< 50% vs. ≥ 50%).

IGRA, interferon γ release assay; IST, immunosuppressive treatment; BCG, Bacillus Calmette–Guérin; SE, standard error; IV, inverse variance; CI, confidence interval; df, degrees of freedom.

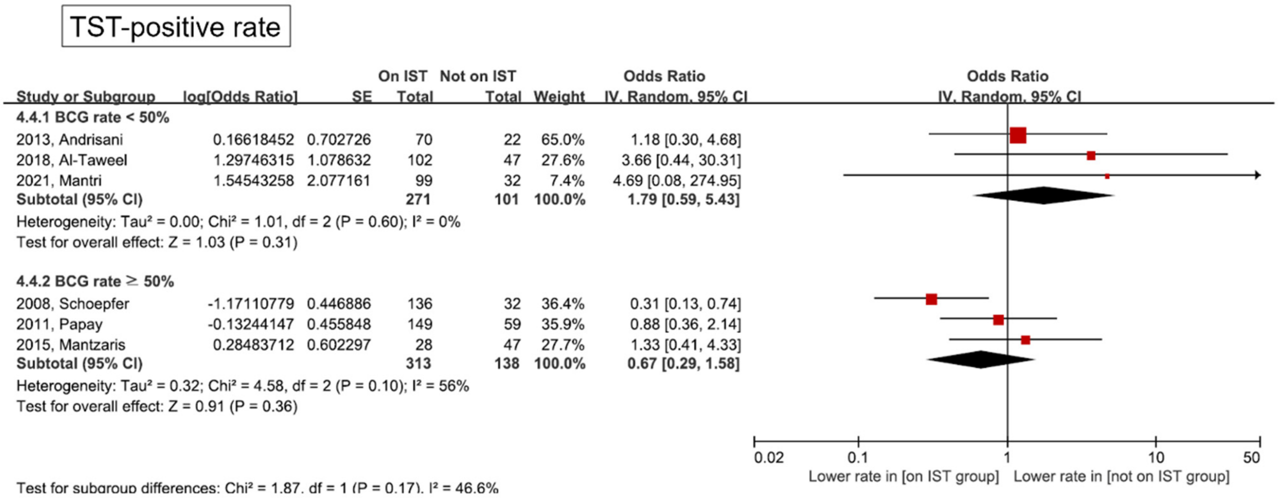

Figure S5. Sensitivity analysis for TST-positive rate according to BCG vaccination rate (< 50% vs. ≥ 50%).
TST, tuberculin skin test; IST, immunosuppressive treatment; TB, tuberculosis; SE, standard error; IV, inverse variance; CI, confidence interval; df, degrees of freedom.
